# A qualitative study of leadership characteristics among women who catalyze positive community change

**DOI:** 10.1186/1471-2458-12-383

**Published:** 2012-05-28

**Authors:** Sara C Folta, Rebecca A Seguin, Jennifer Ackerman, Miriam E Nelson

**Affiliations:** 1 John Hancock Research Center on Physical Activity, Nutrition, and Obesity Prevention, Friedman School of Nutrition Science and Policy, Tufts University, 150 Harrison Ave, Boston, MA, USA; 2 Fred Hutchinson Cancer Research Center, 1100 Fairview Avenue N, Mailstop M3-A410, Room M3-B853, Seattle, WA, USA; 3 Jonathan M. Tisch College of Citizenship and Public Service, Tufts University, Medford, MA, 01255, USA

**Keywords:** Women, Leadership, Public health, Social ecological model, Obesity prevention

## Abstract

**Background:**

Leadership is critical to making changes at multiple levels of the social ecological model, including the environmental and policy levels, and will therefore likely contribute to solutions to the obesity epidemic and other public health issues. The literature describing the relative leadership styles and strengths of women *versus* men is mixed and virtually all research comes from sectors outside of public health. The purpose of this qualitative study is to identify specific leadership skills and characteristics in women who have successfully created change predominantly within the food and physical activity environments in their communities and beyond. The second purpose of this study is to understand best practices for training and nurturing women leaders, to maximize their effectiveness in creating social change.

**Methods:**

Key informant interviews were conducted with 16 women leaders in the public health sector from November 2008 through February 2010. The sample represented a broad spectrum of leaders from across the United States, identified through web searches and through networks of academic and professional colleagues. Most were working on improving the food and physical activity environments within their communities. Questions were designed to determine leaders’ career path, motivation, characteristics, definition of success, and challenges. The initial coding framework was based on the questioning structure. Using a grounded theory approach, additional themes were added to the framework as they emerged. The NVivo program was used to help code the data.

**Results:**

Respondents possessed a vision, a strong drive to carry it out, and an ability to mobilize others around the vision. Their definitions of success most often included changing the lives of others in a sustainable way. Persistence and communications skills were important to their success. The mentoring they received was critical. Challenges included fundraising and drifting from their original mission.

**Conclusions:**

These findings may be used to help develop or inform a model of women’s leadership in public health and to improve the training and nurturance of leaders who promote health in their communities and beyond.

## Background

### Leadership and public health

Historically, obesity prevention efforts have focused on individual behavior change, yielding marginal results and limited sustainability [1,2]. To be effective, solutions will likely need to address the problem more broadly. From a social ecological perspective, it will be necessary to create change at multiple levels, including addressing environmental and policy factors that influence behavior [2-6]. Leadership is one of the major factors in creating change at these levels. For example, leadership has been a critical element in creating policy and shifting social norms around tobacco use and breastfeeding [7].

Leadership has been identified as a key component of community capacity building [8-16], which has emerged as an effective approach for achieving environmental and policy changes to improve health [10,11,17-19]. Within the public health realm, community capacity has been defined as the “characteristics of communities that affect their ability to identify, mobilize, and address social and public health problems” [20]. Notably, enhancing leadership has been a successful strategy for increasing community capacity in underserved populations, including ethnic minority and rural communities [13,15-17], implicating it as a promising approach for reducing health disparities.

In 1998, Goodman *et al.*[8] reviewed the evidence related to leadership characteristics of individuals that contribute to community capacity in the context of public health. This review suggested that successful leaders have a democratic decision-making style; help make it possible for all members of a community to participate; are responsive and accessible; and are well-connected to other leaders [8]. Three studies published subsequently were identified that specifically examine the leadership characteristics related to community capacity. A study of coalition factors that foster community capacity within the Fighting Back Initiative (which addressed substance abuse) used key informant interviews and surveys with project staff, advisory council members, project directors, and steering committee members to identify characteristics associated with greater organizational capacity among the study sites [18]. In this study, higher performing sites had leaders with a more collaborative style, compared to lower performing sites where leaders had a more autocratic style.

In a qualitative study, Goodman [16] explored the components of capacity most relevant to public health initiatives in communities that were predominantly inhabited by racial and ethnic minorities. Group interviews with the core members of each initiative were conducted using an open-ended guide. Cross-site qualitative analysis identified the characteristics of leaders in initiatives that realized successful outcomes, such as improved and expanded health and social services, compared to those in initiatives that failed to achieve goals. Leaders at successful sites were visionary, selfless, persuasive, fearless, and respected. Leaders at sites where initiative goals were not attained were overloaded, overwhelmed, unresponsive, self-interested, and passive. Participatory and team-oriented leadership styles were also found to be more successful than top-down approaches.

A study of the community initiatives to increase physical activity that were part of the Active Living by Design Program defined successful partnerships by positive outcomes such as changes in the community physical environment or in policies related to physical activity [19]. Synthesis of the lessons learned from the 25 communities that participated in the program indicated that leadership was important to success, and that local leaders in the most successful partnerships were visionary, flexible, willing to mentor others, and able to nurture effective partnerships.

Taken together, studies exploring community capacity paint a picture of effective community leaders as visionaries who have the skills to recruit others to a common goal, and to carry out a plan for realizing a vision using a collaborative and democratic leadership style. This is consistent with a transformational leadership model, described by Burns as a model in which leadership is not based on a charismatic personality or access to traditional sources of power, but rather occurs “when one or more persons engage with others in such a way that leaders and followers raise one another to higher levels of motivation and morality” [21]. This type of leadership contrasts both with transactional leadership, which encompasses traditional management practices (setting goals, providing feedback, and exchanging rewards for achievement), and *laissez-faire* leadership, which is characterized by a lack of involvement in managing [22].

Consistent with the studies described above, social and interorganizational networks have been identified as a dimension of community capacity [8]. Explorations of interorganizational work as part of the solution to the childhood obesity epidemic have begun [23]. A body of work has examined an integrative leadership framework in public health, which can be defined as “fostering collective action across boundaries to advance the common good” [24]. The focus is on leadership that cultivates collaboration among multiple levels of the social ecological model and multiple sectors within each level. Integrative leadership scholarship investigates the ways that leadership can foster collaborations that are most effective at creating synergy and change. Crosby and Bryson [25] suggest that eight capabilities are necessary for effectiveness, including an understanding of the social, political, and economic contexts; understanding and deploying personal assets on behalf of beneficial change; nurturing humane and effective organizations; and creating and communicating a shared vision.

### Leadership and women

Although there has been much speculation on the relative leadership styles and strengths of women *versus* men, the literature is mixed [26,27]. In 2002, Eagly and Karau [28] proposed a role congruity theory, suggesting that there is a perceived incongruity between the female gender role, in which communal characteristics (nice and compassionate) are valued, and qualities traditionally associated with successful leadership (assertive and competitive). In a meta-analysis of literature comparing men and women leaders by Eagly *et al.*[29], mainly in the business and educational sectors and defined as supervising or directing the work of others, women were more likely to have a transformational leadership style. The authors suggest that women may favor this style of leadership because it is both effective and consistent with the female gender role, thus providing a way of overcoming role incongruity [29]. Though groundbreaking and highly informative, virtually all of this research comes from sectors outside of public health [29].

### Women leaders in public health

There is a need for studies that examine female leadership specifically within the public health sector, especially with increasing recognition of the importance of changing environmental and policy factors to improve health and the role of leaders in creating these changes. The primary purpose of this study is to identify specific leadership behaviors and characteristics in women who have successfully created change focused predominantly on improving the food and physical activity environments within their communities. In this way, we will begin to elucidate a model of female leadership in public health and determine whether or not it differs from existing models in other sectors. The secondary purpose of this study is to understand best practices for training and nurturing women leaders, to maximize their effectiveness in creating social change.

## Methods

### Design and approach

This qualitative study used semi-structured key informant interviews with women in public health settings. This methodology was chosen because it allowed us to examine their self-described behaviors and practices, their experiences as leaders, and their perceptions of leadership. Our approach was to identify women who were enacting significant changes within their communities and beyond, with an understanding that creating change involves the ability to exert significant influence over others, which is central to leadership. We therefore identified participants based on their actions and impact, so that their leadership characteristics and behaviors would emerge from the data rather than being influenced by a definition of leadership that was based on these factors.

### Recruitment

The research team on this project specializes in community-based research, partnering with organizations such as cooperative extension, state and local governments, and local non-profits to implement and evaluate programming related to physical activity, nutrition, and obesity prevention. To begin the recruitment process, the research team identified academic and professional colleagues across the U.S. who conduct similar community-based research and requested assistance in identifying women leaders age 21 or older, who had been successful in promoting health in their communities and possibly beyond. Two criteria were used as measures of successfully enacting change. The first was demonstration of impact on either the community environment or on policy related to health issues, as determined by the research team. We judged this by factors such as number of people reached, number of years the leader’s organization had been operating and its growth, dissemination of her successful strategies to other organizations, and policies passed by local, state, or national governments that were tied directly to her work. The second criterion for success was achieving recognition through an award for her organization or for her leadership, and/or garnering significant media coverage (in many cases national coverage) that highlighted the impact of her work. We indicated that the leader’s work could be related to any aspect of positive health behavior change. To expand the list of potential interviewees, we additionally conducted a web search using the terms “women” and “leadership”. Since the women initially identified through colleagues were engaging in entrepreneurial activities such as founding a non-profit organization, we added “social entrepreneur” to the web search terms, although we did not limit potential subjects to this category. Through both colleague contacts and the web search, we generated an initial list of approximately forty women. We then gathered information about each potential participant’s organization to ensure that its mission was related to public health. We also gathered additional information about the potential participant and her role within the organization. Through this process we excluded women whose organizations did not clearly have public health as their primary mission or who did not meet our criteria for successful leadership. Our final sample included twenty potential participants, who were sent a recruitment letter or email that described the nature and purpose of the research and requested their participation in the study. Two never responded to the initial invitation for unknown reasons. Seventeen women provided written informed consent, and sixteen completed the interviews. The leader who did not complete the interview had a conflict arise at the scheduled time and attempts to reschedule were unsuccessful. Of the sixteen women interviewed, nine were identified from professional networks and seven from the web search. Women were asked to complete a short questionnaire to obtain demographic information as well as additional background on their community work. All study materials and protocols were approved by the Tufts University Health Sciences Institutional Review Board.

### Interview guide and procedures

One member of our research team (SCF) with expertise in qualitative research methods conducted individual interviews with the sixteen women over sixteen months (November 2008 to February 2010), four in person and 12 by telephone. Once they agreed to participate, leaders were sent an informed consent form and asked to review it, present the research team with any questions, and return it by mail or fax prior to the interview. At the start of the interview, we reviewed the purpose of the study and the confidentiality procedures, and participants were given another opportunity to ask questions.

We used a semi-structured interview guide (see Table 1 for major topics and questions). The entire research team, which included expertise in both community leadership and qualitative methods, provided input on the questioning route. The first question was designed to elicit narratives of the women’s paths that led them to the work they were doing. The purpose was to understand more fully how their current work fit into the context of their lives overall and how they perceived the factors that had shaped them as change agents. A secondary purpose was to establish that we were interested in in-depth responses that reflected their personal meanings and experiences. The rest of the interview guide included open-ended questions designed to explore the women’s thoughts and perceptions about the characteristics of a good leader, steps they had taken to improve their leadership skills, and the role of mentors. In addition, women were asked to describe their motivations, definitions of success, and strategies for overcoming challenges. Interviews lasted 45–60 minutes and were audio recorded. We concluded data collection after 16 interviews. We had achieved reasonable representation in terms of geographic location, age, and race/ethnicity, and preliminary analyses suggested that we had reached a saturation point with respect to key themes [30].

**Table 1:** Interview guide: major topics and questions

**Topic**	**Questions**
Career path	I would like to hear your story about what led you to do the work you’re doing. You can start as early or late in your life as you think is relevant. What started you on this path, and what was the path?
Leadership	When you think about the word “leadership”, what comes to mind?
	Describe the characteristics of a good leader.
	Which of those characteristics do you believe you have?
	Tell me about things you have done over the years to improve your leadership skills. (Prompts: attended a seminar, taken a course, read books on leadership).
	Tell me about any mentors you have had.
Motivation	What specifically motivated you to do this work?
	What keeps you motivated?
Defining success	There are many ways to define success, and many different yardsticks to measure it by. How do you define success in terms of your work? Describe as best you can anything that has helped you become successful, by your own definition.
Challenges	Think back to a time when you faced a significant obstacle or challenge in doing this work. Please describe the obstacle for me.
	How did you overcome it?

### Analysis

Research assistants transcribed recordings of the interviews verbatim. The investigator who had conducted the interviews reviewed the transcripts for accuracy and conducted the primary analysis by coding them. This investigator and two others who had reviewed the transcripts extensively conducted the final analysis by reviewing and discussing the key themes.

Since the first question was designed to elicit narrative, analysis involved examining the responses as a whole (structure, form, and self-representation) rather than breaking them down by coding [31]. Analysis focused on the transformative experiences within the path to the participants’ current work, since these offer information on how to nurture future leaders. The remaining questions were analyzed according to standard qualitative practices, based on grounded theory [30]. The initial coding framework reflected the questioning structure. As coding proceeded, several additional themes emerged and were added to the framework. Coding was therefore an iterative process, grounded in the data. The NVivo qualitative data analysis software (QSR International LTD, version 8.0 for Windows) was used to help code the data.

## Results

The sixteen women ranged in age from 32 to 67 years (Table 2). Twelve were white, three were African American, and one was Asian. Six resided in the northeast region of the United States, while three lived in the Midwest, three in the South, and four on the West Coast. Twelve had founded non-profit organizations. Of the other four, two had served as Executive Directors of their organizations, and the remaining two as directors of a program within a larger organization. Therefore, although we had originally intended to use a broader definition of community change agent, our sample consisted predominantly of women who created change through the establishment of a new non-profit organization.

**Table 2:** Leader characteristics

Race	N
White	12
African American	3
Asian	1
Age in years at interview	Range 32–67; mean 49.8; median 48.0
Geographic location (U.S.)	N
Northeast	6
Midwest	3
South	3
West	4
Highest level of education	N
High school	1
Bachelor’s degree	6
Graduate or professional degree	9
Role in organization	N
Executive Director and Founder	12
Executive Director, not Founder	2
Program Director	2

To preserve confidentiality, we discuss their community activities in very general terms. Seven were working to promote physical activity; four were working on programming to help provide access to healthy, affordable food; and five were promoting health more broadly within specific populations (Table 3). Seven of the women have organizations with national reach; of these, five had started at the community level and expanded, and the other two have major community-level components. Four organizations have regional impact and five are at the community level. Their primary target sub-populations within the community ranged from young children, to families, to older adults. Their organizations range in size (number of official staff) from 1 to 38. All organizations also have a number of associated unofficial (volunteer) staff.

**Table 3:** Organizational characteristics

General organizational focus	N
Physical activity promotion	7
Access to healthy, affordable food	4
Health more broadly	5
Current reach of organization	N
National	7
Regional	4
Community	5
Size (# official staff)*	N
1	5
2–10	7
>10 (range 22–38)	4
Primary target sub-population	N
Low-income families	4
School-age children	3
Those in poverty/homeless	3
Adolescent African American girls	2
All community members	2
“Tween” girls	1
Older adults	1

*Does not include unofficial, volunteer staff.

### The path to leadership

The investigators analyzed the women’s stories for transformative events and experiences. Seven of the women spoke of experiencing a fairly sudden flash of insight, or “epiphany”, that led to their successful community leadership. For one woman, the epiphany involved a sudden sense of her own power and how that might be used in the world (*“Once I’d touched on that kind of strength and internal power, I had to do something with it.”*)(Founder/Executive Director, South, age 48). Three realized immediately that this epiphany would lead them down a difficult path; the insight was therefore almost an unwelcomed addition to their lives (*“This was going to put me out there in a way that is really hard for me, and it still is hard, still is.”*)(Founder/Executive Director, Northeast, age 58). For two, the epiphany took the form of a sudden clear vision of how things could change.

The discussion guide contained no questions about spirituality, yet in seven of the sixteen interviews this subject arose spontaneously. One woman discussed the concrete actions involved in growing her organization entirely within a story arc describing her steady spiritual evolution. Spiritual growth, in terms of a closer relationship with a higher power and greater self-realization and improvement, led to new aspects of her community work and *vice versa*. The work was also a vehicle to support the overall growth of others. Another woman also described her path in mainly spiritual terms, as an ability to listen to her intuition, even though this meant that she usually lacked a concrete plan. She realized this was not a traditional path, and that it may even seem unwise, but she credited it with her success in changing her own life and that of others.

"“I really felt like I was hurling myself off the Grand Canyon. But I chose to take the leap, which I think is a key pivotal point for most people and it really goes against human nature to sort of throw yourself off the Grand Canyon and freefall to possibly your death, into this vast unknown, and that’s where I think most people get stuck. To move forward, to move on to the next level of their spiritual path let’s say. But any way I did it.” (Founder/Executive Director, Midwest, age 47)"

For eight of the women, the change occurred more slowly, either through a series of experiences that raised their awareness and conviction, or through making a change in their own lives that essentially snowballed. For example, one woman’s path was catalyzed by observing her husband’s commitment to being physically active, becoming motivated to do likewise herself, obtaining work in the field, and then expanding her work to a community initiative.

Finally, for one woman, a transformative experience occurred with an invitation into a community that was not her own. The relationships that resulted provided her with motivation for taking action. In her story, she described the ways the community overcame hardship and endurance through the best of human traits: generosity, strength, and optimism. Had she not decided to put considerable energies toward creating an organization to help, she would have been denying these most noble human qualities within herself.

### Leadership characteristics

The model of leadership that the women articulated was characterized by a strong vision along with a total commitment to that vision and an ability to inspire others to share it.

"“I guess I think of a visionary. Someone who is capable of motivating others. Someone who can put their own needs aside for the greater good. Someone who can teach and inspire others to also lead. Who can I guess bring people to action.” (Executive Director, West, age 67)"

The leadership qualities the women generally felt they possessed were related to their ability to carry out their vision. When the investigator asked women which characteristics of a good leader they believed they possessed, persistence and passion emerged as strong themes.

"“…When I first got there they said no fruits and vegetables. They said, ‘they won’t eat it.’ And I said, ‘Who says so? They’re going to eat it! You wait!’ It took me two to three years for them to start eating all those salads, but they did.” (Program Director, Northeast, age 54)"

"“But to me, one of the secrets of my success is not to let obstacles stop me. There are a lot of people who have a lot of good ideas and work very hard. But some obstacles seem insurmountable, and it’s easy to just say, ‘This is too much. It’s impossible. I’ve tried this and it’s impossible.’ That is something that I will not do, would not do…” (Founder/Executive Director, Northeast, age 62)"

Communications skills, compassion, humility, patience, and strength also emerged as key characteristics.

"“I’m very, very good, excellent at public speaking. Telling stories. That has been a gift…I’m so passionate about what I’m doing that I’ve never felt any fear about standing up in front of 10 people, or 1000, just to share the vision for what we’re trying to accomplish here.” (Founder/Executive Director, South, age 48)"

"“I would say the primary [characteristic] is being able to listen. I think that’s something that I’ve gotten better at over the years and I think I was a much worse leader when I was younger. Because I was so convinced that my view and my goals and my plans and vision was right. I would find ways to overcome any obstacles, but that sometimes meant not listening to people who had a different point of view. So I think I’ve learned to be a much better listener, and I think that is really good.” (Founder/Executive Director, Northeast, age 67)"

"“Humility, I think is the biggest thing for me that comes to mind. I think strength, vision, coupled with humility and knowing that however much of a vision you think you have, knowing and understanding that you may not have it all and being open to criticism, being open to input, being open to other ideas.” (Executive Director, West, age 45)"

While many of the women identified these qualities as important to leadership, and particularly their own leadership, they did not necessarily consider themselves to be leaders. Several women noted that they lacked traditional leadership qualities. They were not particularly astute managers, nor were they especially charismatic.

"“Kind of funny, what comes to mind is not me. That can kind of be my fault, is that I don’t always look at myself as a leader.” (Founder/Executive Director, Midwest, age 38)"

"“Well, I am not charismatic and I’ve never been told that I am. I think that would be a good quality.” (Founder/Executive Director, Northeast, age 62)"

None of the questions asked specifically about being a female leader, and only one woman brought up the topic spontaneously. This woman, whose organization addresses physical activity but who had worked on similar issues outside of the public health sector for many years, felt that she had been denied access to opportunities because of her gender. Ultimately, however, this had allowed her to take a less traditional approach which has benefited her.

"“I have felt in the past that opportunities passed me by because they were looking for men…And in some ways maybe that freed me up, so that I…have this unconventional approach…I really did things my own way…When people first think about leadership, they turn to men, and being a female leader has been a little different course.” (Executive Director, South, age 48)"

### Fostering leadership

No clear themes emerged around the use of traditional methods for enhancing leadership, such as workshops and seminars. Many of the women had used these methods and found them to be helpful. Some said that they would like to improve their leadership in these ways, but had no time or money to do so. Others said they had no use for these traditional methods for enhancing leadership.

"“I have looked into seminars and courses, and usually those things cost money, and I haven’t really been willing to spend the organization’s money on those things.” (Executive Director, West, age 45)"

"“I find those things very boring and tedious. It’s embarrassing to say that I don’t have any interest in that…I feel that it’s something you do and you learn about it by doing it.” (Executive Director, West, age 67)"

The women used books as tools more consistently. Although many of the women had read books specifically about leadership, several said that other types of books had been as useful to them including biographies, books on activism, and self-help books.

"“What helps me is I tend to read a lot of autobiographies. And I feel like really taking people that I admire, and know but don’t know in some way, and catching an honest glimpse of their lives and the mistakes they’ve made or the other things they might regret or the things they excelled in, tends to help me.” (Founder/Executive Director, Midwest, age 47)"

"“I don’t know if any of the books have leadership in the title, but books that touch upon leadership or running or managing – not really managing but activism. Books that encompass leadership but aren’t necessarily about how to be a leader.” (Founder/Executive Director, South, age 34)"

Four of the women had used a leadership coach and found that to be helpful.

"“But I have an executive coach; I have had one for the past couple years, through one of our funders, and he has been fantastic for me.” (Founder/Executive Director, Northeast, age 32)"

### Mentoring

Nearly all of the women indicated that mentoring was critical to their success and their growth as a leader. Although this topic was specifically addressed in the interview guide, it often arose spontaneously very early in the interviews. Several major themes emerged in terms of the role that mentors played. One of the major roles was to inspire the women by what they were doing or had done themselves; these mentors included family members, leaders of other organizations, and historical figures.

"“Quite truthfully, my mother, but not from the sense of her being my mother but the challenges that she has dealt with…Just because her hearing is really limited, yet she taught English for, I don’t even know, 20 years. And then continued to move up to administration, and when she retired she was the principal of a school. And so from witnessing that you just keep on going and you don’t take ‘no’ for an answer and you don’t let something that could easily hinder you or give you a reason to change course – you just focus on your course and make it happen.” (Founder/Executive Director, Northeast, age 37)"

"“I think just generally speaking, it sounds awfully cliché, I think a lot of the leadership that was provided during the Civil Rights Movement, particularly from Bobby Kennedy and certainly Martin Luther King. I think their consciousness around the work of movement building is something that I admire…But I would say more from an abstract or conceptual perspective than a sort of attempt to mirror directly in my own behavior.” (Founder/Executive Director, Northeast, age 32)"

Mentors also demonstrated a strong belief in the women leaders, and helped push them to realize their fullest potential, as well as offering specific advice. These mentors often included older women and/or business entrepreneurs, with whom the women had a relatively close relationship.

"“She has seen consistently in me attributes that I haven’t even seen in myself sometimes. I can count on her to push me to do things that I [thought I] couldn’t do. That I couldn’t see. And discover, I can do this.” (Founder/Executive Director, Northeast, age 58)"

"“So he’s been able to really give me a lot of great advice as far as marketing myself, marketing my organization, kind of overcoming that humbleness, which as I said can be a fault at times…So he’s really helped me come out of my shell a lot more.” (Founder/Executive Director, Midwest, age 38)"

### Motivations and definitions of success

The women defined success primarily as the ability to change the lives of others. This is also what kept them motivated to do their work, especially as they faced numerous challenges. The women were not particularly motivated by money or the size of their organizations. Although they appreciated being recognized for their work, prestige did not drive them.

"“So for me, impact is changed lives. Healed bodies, minds, spirits. Especially women who begin to glimpse who they are and go forth. That really matters to me.” (Founder/Executive Director, Northeast, age 58)"

"“It’s hideous to have to try and raise a million dollars every year, six million dollars to build a building you need and if that’s not where your gifts are, it is the tough part. But the offsetting pleasure of seeing people enjoy themselves and benefit from what they are doing…People who were sedentary and now they can get up and take a walk and they make new friends, and now they are hiking, and now they are skiing. It’s a thrilling business and I just love that. To see people blossom in all kinds of ways.” (Executive Director, West, age 67)"

"“But I would say that more concretely is when I actually see a change in the individuals that we work with. So to our youth training program, to actually see the penny drop, to see people shift in terms of how they view food as a social justice issue, in terms of how they suddenly see their own role in the world, in terms of not just having to be receivers of whatever is handed out to them, but being an agent of change. I think that’s what keeps me motivated.” (Executive Director, West, age 45)"

The women’s other measures of success included having changed policy and supporting a healthy, thriving, sustainable organization.

"“An then just little successes like we work on things that are related to policy change or environmental change that changes the conditions in which people live, to make the healthy choice the default option, the easier choice, the thing that just happens. So when I get those little successes, that really makes me feel like, wow, if I hadn’t done that then that wouldn’t have happened. That’s pretty cool!” (Program Director, Midwest, age 53)"

"“The big, hairy idea of success is that, you know, we’ll build a movement, and we’ll do a national direct service program, and then on the shoulders of that, a training program that serves even more kids, and then on the shoulders of that, an advocacy and policy sort of approach that creates a system where the expectation is that kids have access to healthy, safe play.” (Founder/Executive Director, West, age 44)"

"“I would say there would be some measure of success when I am able to leave and other young people from the neighborhood are running the work, which is what we’re planning, that process and that transition now. When that happens, then I’ll feel like yes, I did something right.” (Executive Director, West, age 45)"

The women identified the support of others, particularly family members, as an important factor in achieving success as they defined it.

"“I would say my family. The support of my husband and my own children. I think that’s what keeps me going. They remind me of – when I’m feeling maybe a personal failure, or worried, or whatever about anything with [program], they’re the ones that remind me to keep going and that great things are happening.” (Founder/Executive Director, Midwest, age 38)"

### Challenges

The women faced a common set of challenges. Not surprisingly, obtaining adequate funding was one of these.

"“It’s not easy, especially at the beginning, especially in the first 3 or 4 years to go and say, ‘Please give me a lot of money, and this is my idea, I’m going to make it work.’” (Founder/Executive Director, Northeast, age 62)"

A shared strategy for overcoming this challenge was conducting thorough research. This allowed the women to demonstrate their mastery of the issue, and it also allowed them to find the best ways to position their cause so that it would resonate with potential funders.

"“I know how to approach people and do my elevator speech. I understand what I’m doing. I’ve done a lot of research…” (Founder/Executive Director, West, age 63)"

The women identified organizational growth as another common challenge. Success brought with it difficulty in staying true to an original mission, as well as a changing culture, as the programs and organizations grew from a few highly dedicated people to a more organized (and sometimes hierarchical) structure.

"“It was not easy. So there were some times where I was afraid we were getting a little too far from our mission. Lots of sleepless nights. Oh my gosh. Trying to figure out how to bring it back.” (Founder/Executive Director, South, age 48)"

Women noted that overcoming challenges often involved being able to recognize their own limitations and bringing in personnel to complement their skills. This meant letting go of some control. While initially this may have been a painful process, ultimately it fit with the women’s own definitions of leadership, which included the empowerment of others.

"“I would say that would be the defining characteristic of the way most obstacles around here get solved, is that I get out of the way (laughs)… I had to come to terms with the fact that I’m a great leader, but I’m not a particularly astute manager. I had to really recognize that if we were going to achieve scale, that we needed to bring in folks who really focused on managing others. That was hard… So, yeah, I think stepping out of the way and letting other people really play to their strengths.” (Founder/Executive Director, West, age 44)"

## Discussion

In the past decade, there has been increasing recognition that improvement in public health, and in the food and physical activity environments specifically, can and will occur through community-level change, and that leadership plays a key role in bringing about this change [7]. When leaders are an integral part of their communities, they bring a deep understanding of what is most needed and feasible within their own specific contexts. This study helps elucidate that these successful women leaders share the transformational model of leadership and suggests ways to train and nurture other women leaders so that they will be maximally effective in creating change.

### Women leaders in public health: Comparison with existing literature

The model of leadership that emerges from this work is consistent with previous work [8,16,18] and with the transformational leadership model. The latter consists of four components [32]. The first is Idealized Influence, which has two aspects: characteristics such as high levels of integrity that garner the respect, trust, and admiration of followers, and behaviors such as communicating a collective mission and purpose. This aligns with the leaders’ descriptions of themselves as visionaries with the ability to communicate their vision. The second component of the model is Inspirational Motivation, which involves motivating followers by expressing enthusiasm and optimism for a potentially improved future state. This is reflected in the strong theme that emerged around inspiring others around a vision. The third component is Intellectual Stimulation, which involves stimulating creativity and finding new ways to address old problems. Each of our leaders had developed a unique approach to addressing a community issue, and most had founded a non-profit organization to implement their new approach. However, an aspect of this component is the ability to stimulate creativity in others, which was not evident in our results. The final component of the transformational leadership model is Individualized Consideration. Leaders foster follower’s growth by attending to individual needs and serving as a coach or mentor. Although the responses of leaders in our study provide no direct evidence for this component, they did emphasize communications and listening, and defined success in part by whether they had changed the lives of the people they worked with in addition to those served by their organization.

Most of the leaders’ non-profit organizations worked across sectors, involving government and/or business to make environmental and policy changes. Although research on integrative leaders is relatively new, there is a growing consensus on the qualities and behaviors possessed by these leaders, some of which emerged in this study. Consistent with the transformational leadership model, these qualities include creating and communicating a shared vision [25,33-35], being innovative, taking risks, and expressing dissatisfaction with the status quo [33,36]. Effective integrative leaders also appear to have highly developed relational skills, with an ability to establish shared meanings, listen actively, build trust, and manage conflict [33,37], characteristics that did not emerge as strong themes in this study. Some evidence suggests that leaders may exhibit more relational skills when leading within collaborations and networks than when leading solely within their own organizations [37]. Given the questioning structure in this study, it is likely that participants’ responses reflected their organizational rather than their collaborative leadership. Future studies may specifically investigate the role of women leaders in cross-sector work related to improving the food and physical activity environments, as well as the leadership characteristics and behaviors related to this work.

The results of this study have implications for the role of gender in leadership style in a sector outside of business or education. These women, who were largely able to define their own organizational role, gravitated toward a leadership style (transformational) that does not conflict with gender roles. This is consistent with research that suggests that certain gender-role specific traits remain despite organizational role [38]. However, it is also possible that these leaders were reflecting general trends toward a more transformational style, considered to be more effective in the current culture [32]. Further research in this area will help elucidate the relative importance of organizational *versus* gender roles in leadership style in the public health sector.

Several of the self-described characteristics that emerged in this study, such as compassion, humility, and patience, are consistent with a communal style that is more stereotypically female. However, the leaders also described themselves as being persistent and passionate, qualities related to confidence and ambition, which are more stereotypically male as well as more consistent with cultural stereotypes of leadership [39]. Similar themes emerged in a qualitative study with high achieving African American and white women across ten fields [40]. Although these women described themselves as possessing several stereotypically male traits, such as persistence, they did not appear to deny or downplay their more stereotypically feminine qualities in order to succeed.

### Training and nurturing future leaders

Our results suggest ways to train and nurture women leaders in public health. It is important to first understand the best ways reach these leaders. It would be important to differentiate any offered workshops and to emphasize in promotional materials that the investment of time and money would translate to increased effectiveness in changing lives. Our results also suggest that a book may be an attractive leadership tool, and that it might be most compelling if it emphasized *purpose* – to provide both inspiration and practical support in effectively carrying out a vision for creating change – rather than on traditional leadership.

Community change leadership tools could also include information and ideas for overcoming the most common challenges in this arena: fundraising and organizational growth. With respect to fundraising, several of the women described the benefits of doing detailed research on their topic and having the evidence at hand. Skill-building around database searches and literature reviews (areas that academics are particularly well-suited to assist with) would streamline this process. The tool might also include information on the stages of organizational growth, the anticipated challenges at each stage, and lessons learned from other leaders on how to best address those challenges.

Our results also suggest ways to best nurture women leaders in public health. Receiving mentoring was critical to the women leaders in our study. A leadership tool could provide information on identifying appropriate mentors to fulfill several key roles. The tool could also provide support for self-care, which might include putting a robust personal support network in place, encouraging spiritual growth (if appropriate), and making the most of motivating factors – by, for instance, deliberately collecting stories of the lives that had been changed through their work.

### Limitations and implications

As with any type of qualitative research, the generalizability of these findings may be limited. However, we were able to achieve diversity in terms of ethnicity, geographic location, and types of community health issues addressed. We believe that the results generalize reasonably well to women leaders who are creating change within their communities to improve public health.

This study was conducted by female researchers on female leaders. We attempted to minimize any potential effects of our own biases by adhering to the questioning route as strictly as was reasonable in a qualitative study. We also conducted the analysis using software as a tool so that the data could be examined more objectively, and we worked as a team so that interpretation did not rely on a single person. However, results may still reflect our own biases. It is also possible that the leaders responded to us differently than they would have to male researchers.

In this qualitative study, leadership characteristics are measured by self-report. The study might have been strengthened by obtaining feedback from staff and volunteers as well. Finally, this study is limited by the lack of a comparison group, which would be comprised of similar community leaders who had not met our definition of success. Because of this, we are not able to conclude that the characteristics that emerged are specifically associated with success. However, we report the characteristics that emerged strongly and consistently among a diverse sample of successful women leaders. Furthermore, these characteristics are consistent with those observed in other studies of successful community leaders.

This research was designed to elucidate women’s leadership characteristics, but not to compare and contrast them with men’s. With these results serving as a base, future studies may include this type of analysis, for example comparing results of men and women in similar public health leadership positions on the validated Multifactor Leadership Questionnaire, which is designed to distinguish between transformational, transactional, and *laissez-faire* leadership styles [41]. Further studies may also involve creating and evaluating a leadership tool designed to train women leaders in public health.

## Conclusions

This research confirms the important role that community leaders play in improving public health. The sixteen women interviewed have made important contributions, from the development of physical activity programs for children to the creation of coalitions that have ultimately affected national policy on active transportation. Collectively, they have helped to improve the health of thousands of people. With the increased use of social ecological approaches to address the obesity epidemic as well as other major public health issues, leadership is likely to be a key factor, and including a leadership component may greatly enhance future interventions. This research helps elucidate a model of public health leadership for women, as well as suggesting ways to best train and nurture leaders so that they may be maximally effective at creating change within their communities and beyond.

## Competing interests

The authors declare that they have no competing interests.

## Authors’ contributions

SCF carried out the interviews and was responsible for the primary analysis. All authors participated in the design of the study, participated in analysis by reviewing transcripts and key themes, and all have read and approved the final manuscript.

## Pre-publication history

The pre-publication history for this paper can be accessed here:

http://www.biomedcentral.com/1471-2458/12/383/prepub
